# Effects of Climate Change on Chlorophyll *a* in the Barents Sea: A Long-Term Assessment

**DOI:** 10.3390/biology12010119

**Published:** 2023-01-11

**Authors:** Vladimir G. Dvoretsky, Veronika V. Vodopianova, Aleksandra S. Bulavina

**Affiliations:** Murmansk Marine Biological Institute of the Russian Academy of Sciences (MMBI RAS), 183010 Murmansk, Russia

**Keywords:** phytoplankton, climatic fluctuations, warming, Arctic, Barents Sea, generalized additive models

## Abstract

**Simple Summary:**

Phytoplankton and other algae form the bases of food webs in aquatic systems as they convert solar energy into chemical compounds that are consumed by the higher trophic levels. Through the photosynthesis process based on chlorophyll *a* (Chl-a), phytoplankton uses sunlight to take carbon dioxide and release oxygen. Chl-a is a good indicator of phytoplankton biomass and may be used to detect changes in marine ecosystems due to environmental fluctuations. Dramatic climatic changes have been observed in the Arctic during the past decades. The study aimed to give up relations between Chl-a and climatic factors in the Barents Sea. We found an increase in Chl-a over the last four decades, with maximum values in warm periods. High temperature and a decline in sea ice extent were associated with greater Chl-a concentrations. Atmospheric processes estimated through the North Atlantic Oscillation indices strongly affected the surface water temperature, salinity, and Chl-a. Mapping of the Barents Sea showed high concentrations of Chl-a associated with the ice edge in spring and coastal waters in summer. Our study suggests that spatial, seasonal, and temporal variability in Chl-a is controlled by temperature changes, ice extent, and global atmospheric circulation and may be used for future investigations dealing with climatic forcing in the Arctic marine systems.

**Abstract:**

The Arctic climate strongly affects phytoplankton production and biomass through several mechanisms, including warming, sea ice retreat, and global atmospheric processes. In order to detect the climatic changes in phytoplankton biomass, long-term variability of chlorophyll *a* (Chl-a) was estimated in situ with the changes in the surface sea temperature (SST) and salinity (SSS) in the Barents Sea and adjacent waters during the period of 1984–2021. Spatial differences were detected in SST, SSS, and Chl-a. Chl-a increased parallel to SST in the summer-autumn and spring periods, respectively. Chl-a peaks were found near the ice edge and frontal zones in the spring season, while the highest measures were observed in the coastal regions during the summer seasons. SST and Chl-a demonstrated increasing trends with greater values during 2010–2020. Generalized additive models (GAMs) revealed that SST and Chl-a were positively related with year. Climatic and oceanographic variables explained significant proportions of the Chl-a fluctuations, with six predictors (SST, annual North Atlantic Oscillation index, temperature/salinity anomalies at the Kola Section, and sea ice extent in April and September) being the most important. GAMs showed close associations between increasing Chl-a and a decline in sea ice extent and rising water temperature. Our data may be useful for monitoring the Arctic regions during the era of global changes and provide a basis for future research on factors driving phytoplankton assemblages and primary productivity in the Barents Sea.

## 1. Introduction

Phytoplankton assemblages in the World Ocean strongly depend on climatic fluctuations, availability of nutrients, and light [1]. Changes in phytoplankton stocks and composition can affect total productivity and carbon storage in pelagic ecosystems [2].

The Arctic Ocean has experienced clear fluctuations in sea surface temperature at interannual to multidecadal scales due to the impact of the North Atlantic Oscillation [3,4]. Moreover, significant warming processes have been documented in the Arctic as a result of global climate change, and this trend is predicted in the upcoming decades [5,6,7]. Satellite observations and remote-sensing data on averaged chlorophyll *a* (Chl-a) and productivity indicated different trends and relationships with environmental variables in the upper open ocean systems, including Arctic regions [8,9,10]. The impact of rising temperatures on ocean productivity is currently considered one of the major challenges for marine science. 

**Figure 1:**
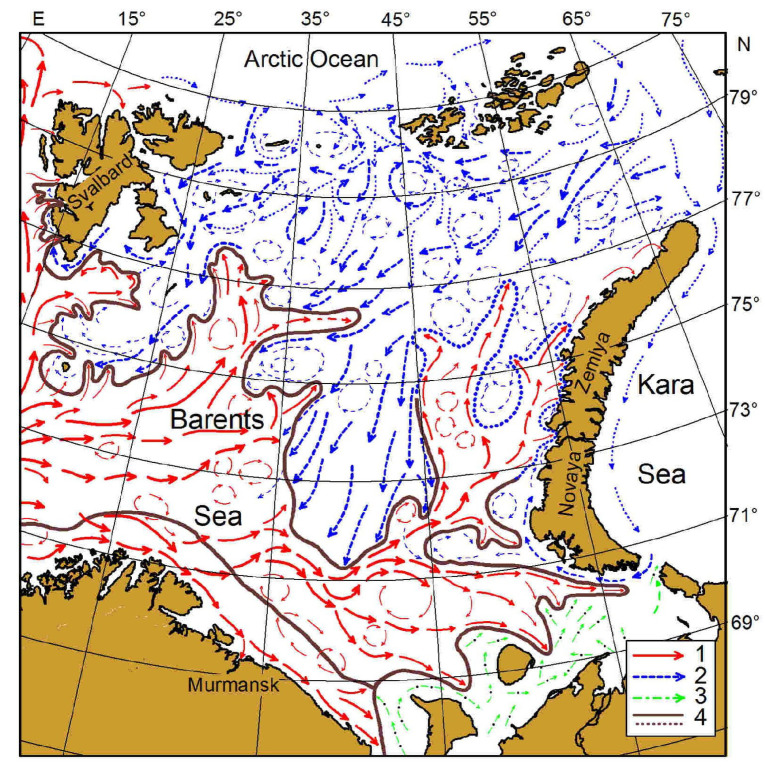
Study area, Barents Sea, and main currents: 1—warm flows; 2—cold flows; 3—White Sea and Pechora Sea flows; 4—boundaries of frontal zones [11,12].

The Barents Sea represents a shallow shelf region with the western boundary defined by the shelf break towards the Norwegian Sea, the eastern boundary confined by Novaya Zemlya, the southern boundary located off the Kola Peninsula, and the northern boundary limited by the continental shelf break towards the deep Arctic Ocean (Figure 1). 

It has an area of approximately 1.6 million km^2^ with an average depth of 230 m and a maximum depth of about 500 m [13]. Being located between 70° and 80° N, it is characterized by strong seasonal variations in light regime from 24 h of darkness in polar winter and 24 h of continuous sunlight in summer [14]. The oceanography and ecosystem dynamics in the region are significantly influenced by warm Atlantic waters flowing from the Norwegian Sea to the west and cold Arctic waters advected from the north [15,16]. Three main types of water masses can be distinguished in the Barents Sea based on their hydrological properties: Atlantic Water (AW; temperature 1–8 °C, salinity 34.90–35.15 psu), Arctic Water (ArW; temperature −1.8–2 °C, salinity 32.00–34.80 psu), and Coastal Water (CW; temperature −1.8–9 °C, salinity 30.00–34.90 psu) [11,13].

Extensive seasonal fluctuations in ice coverage are detected in the Barents Sea, especially in the eastern areas [16]. The ice extent in the Barents Sea has dropped by 60% over the last two centuries [17]. The extent of the ice cover depends on the air and water temperatures, the North Atlantic Oscillation (NAO), oceanic circulation, and the river run-off from land [18,19,20]. The Barents Sea is considered one of the most productive regions in the Arctic. The annual production in the region varies between 20 and 200 g cm^–2^, with the greatest values in AW. The average total annual primary production is approximately 70–100 g cm^−2^ [14,19]. In general, in ArW, primary production is largely due to the spring bloom, while in AW, a relatively large part of primary production occurs in the summer and autumn [11]. Thus, the annual primary production in AW is approximately three times, and new production is two times higher than that in ArW [14,19]. 

In the AW domain, the phytoplankton growth and development during spring are controlled by thermocline formation due to strong water stratification, while in ArW, the timing of the ice edge phytoplankton bloom is defined by the stability of the water column from melting ice [19,21]. Recent studies have proposed earlier phytoplankton peaks in the Arctic domain of the Barents Sea [22,23]. Zooplankton abundance and stocks show clear interannual changes caused by both top-down and bottom-up processes [24,25,26,27,28]. Copepods of the genus *Calanus* represent the major part of the total zooplankton in the Barents Sea, and they comprise 75–80% of the total biomass [29]. Zooplankton is an important food source for pelagic planktivorous fishes, including young herring, capelin, polar cod, and pelagic 0-group of cod and haddock [11,14]. Some studies have reported an expansion of boreal zooplankton and fish northward and eastward, likely due to the improved habitat conditions, advection with AW, and warming processes [6,15,16,30]. Alterations in biological systems of the Barents Sea, namely, shifts in the structure and phenology of phyto- and zooplankton, a decrease in phytoplankton cell size, and an increase in primary production, may be indicators of possible future changes in the Arctic [9,23,27,31].

Chl-a concentrations are often used as a good proxy to estimate phytoplankton biomass in the marine environment [32,33,34]. SeaWiFS and MODIS satellite imageries provide data on the Chl-a content in the surface layers. Ocean color remote sensing requires the development of accurate algorithms and high-quality input data, which are not always available [35,36], especially in high-latitude regions. Arctic-specific algorithms can be applied successfully when parameterized correctly [37]. However, large solar zenith angles, persistent cloud cover, and high riverine fluxes into coastal waters make it difficult to adequately apply satellite-derived data in the Arctic [37]. As a result, there must be some errors in the data provided by the standard algorithms of SeaWiFS and MODIS [38]. The errors are highest at low Chl-a concentrations and close to zero at Chl-a concentrations above 0.5 mg m^−3^ [38]. Therefore, in situ Chl-a measurements must be considered a preferable source for accurate Chl-a estimations. Nevertheless, remotely sensed measures of Chl-a may have some advantages when studying the long-term dynamics of phytoplankton biomass over larger spatial scales in various regions [37].

There was large interannual variability in the satellite-derived surface Chl-a concentrations and magnitude of the spring bloom in the Barents Sea over the 1998–2017 period [39]. Strong differences in the Chl-a content were registered from place to place and seasonally. In the Barents Sea, there is a regular seasonal cycle in Chl-a [40,41,42]. The maximum occurs during the spring period [41]. In general, throughout summer, Chl-a values tend to remain lower, although there may be some fluctuations; then, during autumn, there is a small rise in Chl-a [11,14,19]. Minimum Chl-a values are encountered during winter. A clear spatial pattern of Chl-a is also evident, with a higher average spring peak for the southern and central regions [14]. The start of the spring bloom is strongly associated with the timing of the peak bloom [40]. Significant positive relationships between ice-free conditions and satellite-based net primary production have been reported [11,39]. Recent phytoplankton dynamics in the Barents Sea have been changed, and this process is controlled mainly by bottom-up processes [39]. Thus, responses of Arctic phytoplankton to environmental fluctuations are well recognized [39,40,41,42], but there is a continuing need to distinguish long-term trends caused by climatic variability changes. To better understand phytoplankton dynamics in the Barents Sea in relation to climatic fluctuations, further research based on real (in situ) Chl-a measurements is strongly needed. 

The aim of the present study was to analyze long-term variations in the surface Chl-a in the Barents Sea with special attention to large-scale climatic factors. We hypothesize that in situ Chl-a estimations would be associated with variations in environmental factors. To test our hypothesis, we examined links between the surface Chl-a and water temperature, salinity, indices of global atmospheric circulation, temperature/salinity anomalies, and sea ice extent. Using generalized additive models, we studied which factors were responsible for Chl-a variability in the region.

## 2. Materials and Methods

### 2.1. Sampling

**Figure 2:**
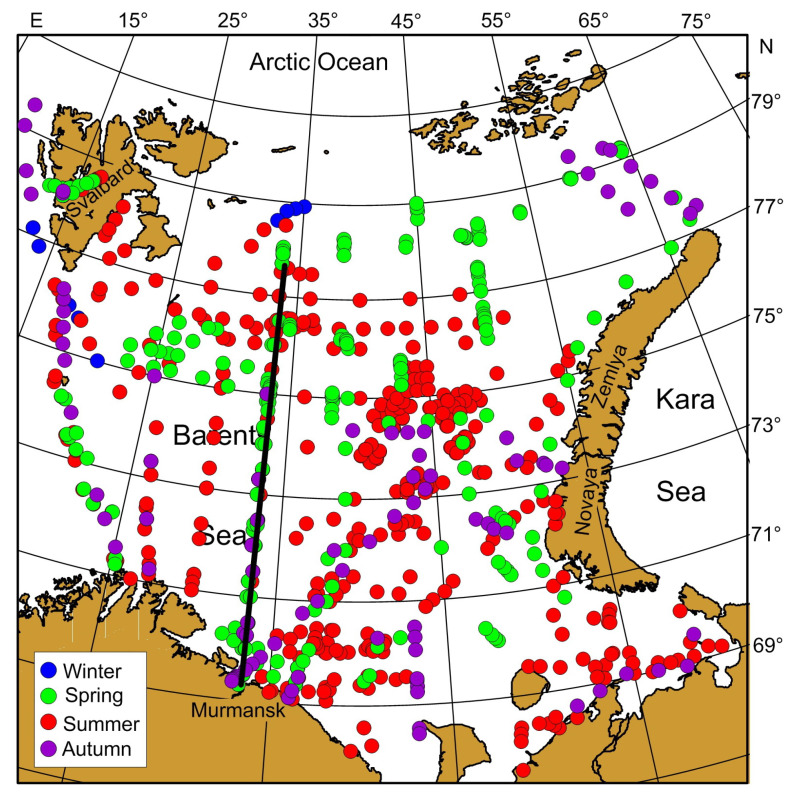
Sampling locations in the Barents Sea during 1984–2021. Solid black line indicates the Kola Section (69°30′–77°30′ N, 33°30′ E).

In situ data were obtained during 27 cruises in the Barents Sea and adjacent Svalbard waters during the period 1984–2021 (Table 1, Figure 2). A total of 803 stations were sampled in the surface layer. Chl-a samples were collected with Niskin bottles mounted on a CTD system (1993–2021) or a bottle attached to a steel rope (1984–1988). Seawater samples (5 L) were filtered onto GF/F filters and frozen onboard. The samples were analyzed in the laboratory of MMBI using 90% acetone as an extracting agent. Chl-a concentrations were determined fluorometrically according to standard procedure during 1984–1993 [43]. In the period 2006–2021, the Chl-a contents were measured using a Nicolett Evolution 500 spectrophotometer (Spectronic Unicam, Great Britain) calibrated with commercially purified Chl-a [44]. Surface temperature and salinity were recorded at each station with a Sea-Bird Electronics SBE 19plus V2+CTD (Conductivity-Temperature-Density) sonde in the period 2006–2021. From 1984 to 1993, temperatures were recorded using manual mercurial thermometers while salinity was measured chemically according to a standard procedure [43,45].

### 2.2. Environmental and Climatic Indicators

Seven environmental measures were selected in order to analyze the possible influence of climatic fluctuations on the Chl-a pattern in the Barents Sea: the North Atlantic Oscillation indices (annual and winter), temperature and salinity anomalies (0–200 m) at the Kola section (70°30′–72°30′ N, 33°30′ E), annual sea ice coverage (% of the total area), sea ice extent (km^2^) in April and September. We also used local environmental indices (SST and SSS—in situ sea surface temperature and salinity, respectively) to reveal their possible impact on Chl-a variability.

The NAO is the most important parameter of atmospheric variability over the North Atlantic and the adjacent Arctic, including the southern and south-western Barents Sea. The NAO index assesses the strength of the zonal flow across the North Atlantic and consists of the pressure difference between Iceland and the Azores [46]. The NAO index reflects the oscillation of a large-scale anomalous pressure pattern or large-scale meridional exchange of atmospheric mass. The annual NAO index used in our work was calculated as the difference between the normalized sea-level pressure in Ponta Delgada (Azores archipelago) and Stykkisholmur/Reykjavik (Iceland). The winter NAO index was calculated based on the data for the period December (previous year) and January–February current year); the data were provided by the Climate Analysis Section, NCAR, Boulder, USA [47,48].

The Kola Section is a standard oceanographic transect located between 69°30′ and 77°30′ N in the Barents Sea. Oceanographic studies at this transect have been monitored during the past decade [11,49,50]. Temperature and salinity anomalies calculated at stations 3–7 are the longest time-series of oceanographic data for the Barents Sea [49]. These anomalies are considered good indicators of climatic variability in the region [16]. Data on the temperature and salinity anomalies were downloaded from the repository of the Polar Research Institute of Marine Fisheries and Oceanography (PINRO) (http://www.pinro.vniro.ru (accessed on 26 June 2022)) and updated from recently published sources [15,16].

Annual sea ice extent data expressed as percentages of the total sea area were provided by the Working Group on the Integrated Assessments of the Barents Sea (WGIBAR) [16]. Monthly ice concentrations were used to calculate the sea ice extent in the Barents Sea (rectangle demarcated by latitudes 72° N and 82° N and longitudes 10° E and 60° E). Within this rectangle, grid cells of 25 km × 25 km each were defined as ice covered if 15% or more of the area is covered by ice [16,51]. In most years, the maximum extent of sea ice cover in the Barents Sea is in April, and the minimum is in September. To assess the sea ice extent in April and September (km^2^), we used data from the Norwegian Polar Institute [51].

### 2.3. Statistical Analyses

All data were divided into 5 five groups: spring, summer, autumn, and winter, and combined for all seasons. The normality of the environmental and Chl-a data was checked with the Shapiro–Wilk test. In situ values of Chl-a, SST, SSS, and climatic parameters were found to be normally distributed (Figure S1). Therefore, raw datasets of most variables were used as non-transformed in the analyses except for the ice indices. Annual, April, and September ice extents were lg-transformed in order to stabilize the variance. We studied seasonal effects on the in situ field data by testing for differences in the mean values of SST, SSS, and Chl-a using one-way ANOVA and Tukey’s post hoc tests. 

Generalized Additive Models (GAMs) with climatic indicators as continuous variables were applied to investigate temporal variability in Chl-a in the Barents Sea. GAMs have been found to be a good tool for analyzing non-linear and non-monotonic relationships between a response variable and fitted predictors [52]. Shortly, a GAM represents a set of non-parametric and semi-parametric regression techniques to explore relationships between response and predictor variables. GAM can be used without any prior assumption on the functional form linking the two sets of variables, and these relationships are modeled with smoothed functions. The model relates a univariate response variable, Y, to some predictor variables, x_i_. An exponential family distribution is specified for Y (for example, normal, binomial, or Poisson distributions) along with a link function g (for example, the identity or log functions) relating the expected value of Y to the predictor variables via an equation such as [52]:

g(E(Y)) = β_0_ + f_1_(x_1_) + f_2_(x_2_) + ⋯ + f_n_(x_n_).

In our study, the response variable (Chl-a) might demonstrate linear or non-linear relationships with the explanatory variables (SST, SSS, and climatic indicators). Therefore, we used GAM with both normal and Poisson distributions along with an additive link function (identity) to predict Chl-a fluctuation in relation to environmental variables. Cubic regression splines as the smoothing functions were applied in GAMs with the smoothness determined by estimated predictive accuracy. General algorithms added in the models used any regression-type smoothers as partial residuals. The partial residuals (Chl-a) remove the effects of all other variables; therefore, the Chl-a can be used to model against the effects of climatic predictors. Interannual trends of SST, SSS, and Chl-a (influence of year as a model predictor) were studied with GAMs and linear regressions. We used only values collected at the Kola Section (69°30′–74°30′ N, 33°30′ E) to avoid spatial influence on the data. Data for this site covered most of the study period and can reflect relevant temporal patterns.

The GAMs were performed using StatSoft STATISTICA 10 software (www.statsoft.com (accessed on 12 August 2022)). Mean values were calculated with standard errors (±SE). We applied an interpolation to determine the SST, SSS, and Chl-a using ordinary kriging (spherical semi-variogram model). The contour maps for SST, SSS, and Chl-a were created using MapViewer 6.00 (Thematic Mapping System, Golden Software Inc., Golden, CO, USA).

## 3. Results

### 3.1. Temporal Variations in Climatic Indicators

**Figure 3:**
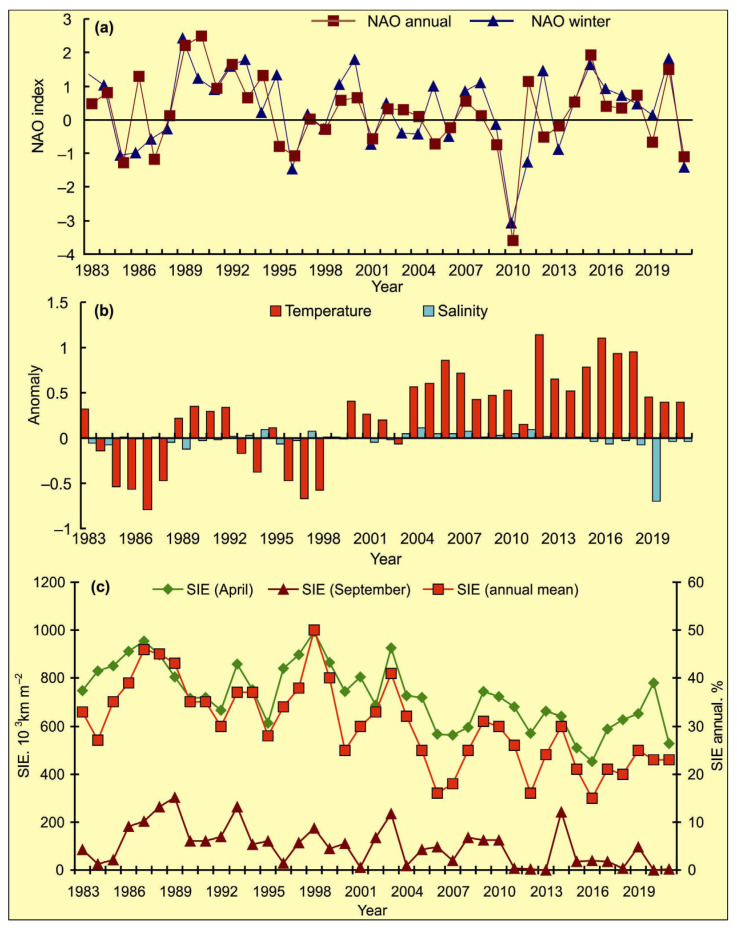
Variations in the climatic indices in 1983–2021. (**a**)—annual and winter (DJF) NAO indices in 1984–2021 [53]. (**b**)—annual temperature and salinity anomalies in the 0–200 m layer at the Kola Section. St. 3–7—Murman Current [15,16]; (**c**)—sea ice extent (SIE) in April and September, and annual mean SIE in the Barents Sea expressed as a percentage of the total sea area [15,16,51].

The NAO index varied during the period 1984–2021, and the long-term trend was stationary around zero. Some specific periods of higher or lower modes of NAO were evident (Figure 3a). A general increase in the NAO indices occurred from 1987 to 1994. The periods of anomalously positive events can be noted in the following NAO series: 1989–1994, 1999–2000, and 2014–2020 (Figure 3a). 

Temperature anomalies at the Kola Section were found to be positive during 1989–1992, 2000–2002 and since 2004, and were generally typical for warm years (Figure 3b). Salinity anomalies in CW and AW at the Kola Section were close to zero from 1990 to 2018, with slightly positive values from 2004. The highest salinity anomaly was recorded in 2019, while in the next years, salinity anomalies were near 0 (Figure 3b). 

The percentage of ice cover was lower relative to mean multi-year values in the following periods: 1983–1984, 1991–1996, 1999–2001, and since 2004 (Figure 3c). In April, the ice extent showed high values during the years 1987, 1998, and 2003 whereas low values were registered during 2006, 2007, 2008, 2012, 2015, 2016, and 2021 with the lowest ones in 2015, 2016, and 2021. In September, the highest values were observed in 1989, 1993, 1998, 2003, and 2014, while the lowest estimations of ice cover were observed in 1984, 1996, 2001, 2004, 2011, 2012, 2013, 2018, 2020, and 2021 (Figure 3c). 

### 3.2. Spatial, Seasonal, and Temporal Variations of SST, SSS and Chl-a

**Figure 4:**
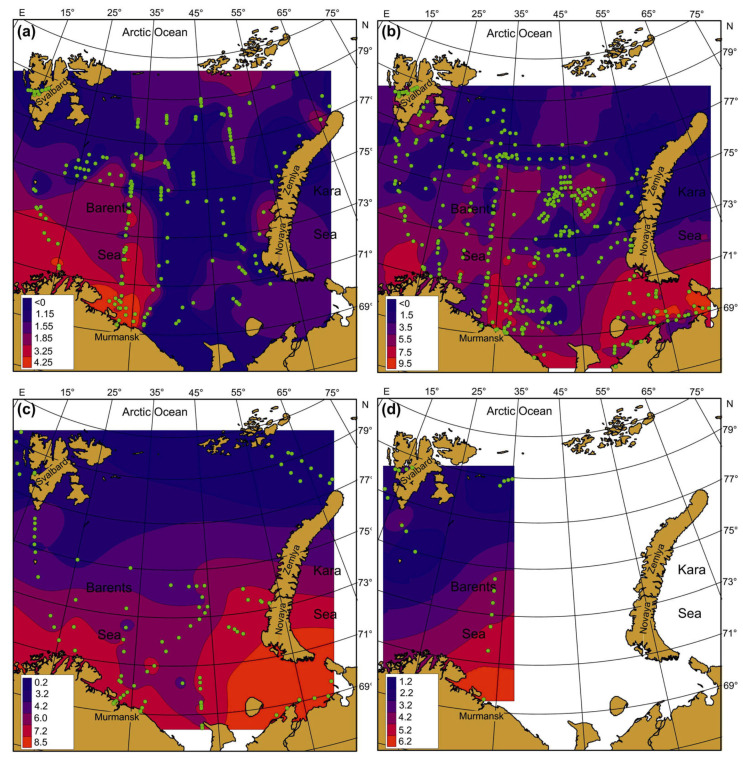
Averaged SST (°C) in the Barents Sea, 1984–2021. (**a**)—spring; (**b**)—summer; (**c**)—autumn; (**d**)—winter. Points indicate the location of sampling stations.

**Figure 5:**
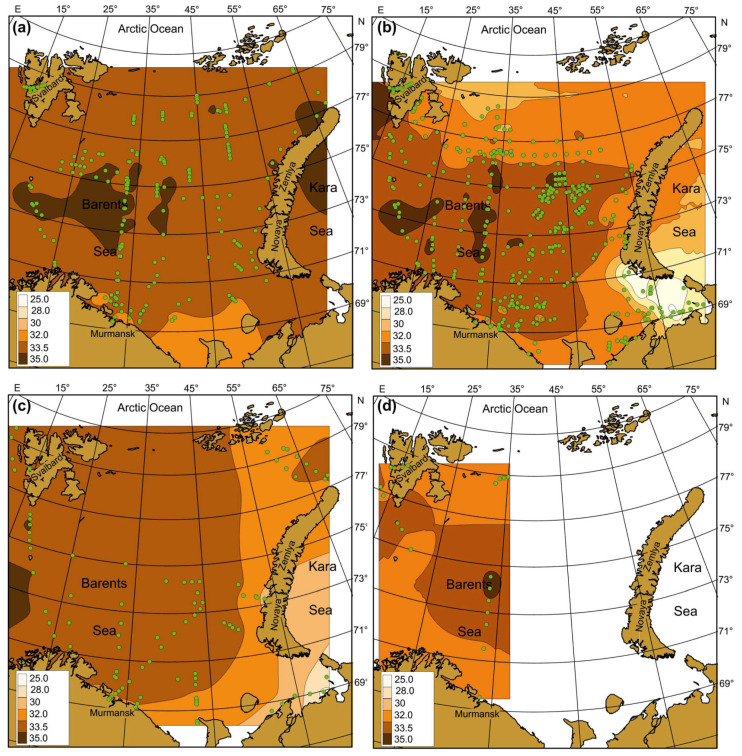
Averaged SSS (psu) in the Barents Sea, 1984–2021. (**a**)—spring; (**b**)—summer; (**c**)—autumn; (**d**)—winter. Points indicate the location of sampling stations.

**Figure 6:**
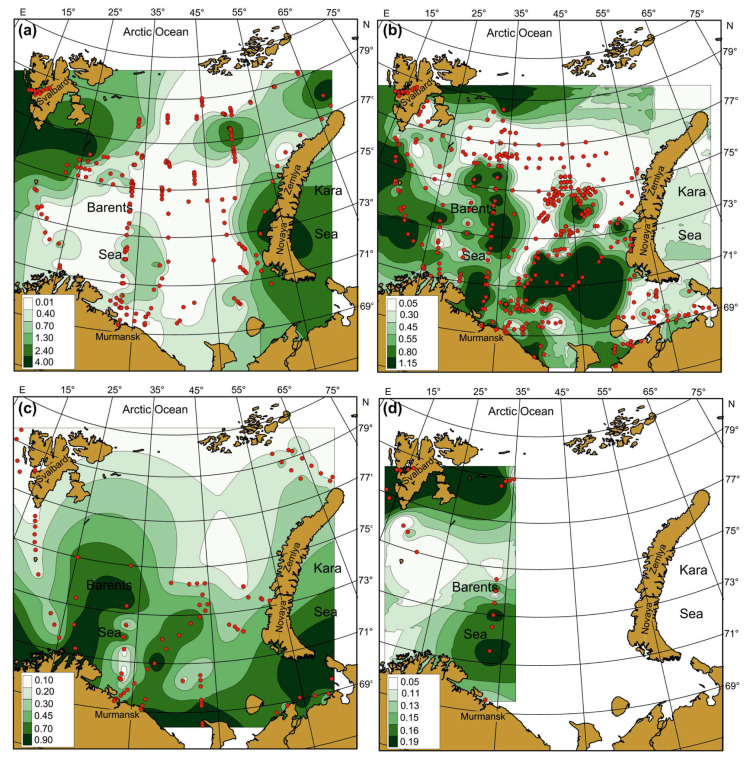
Averaged Chl-a (mg m^–3^) in the Barents Sea, 1984–2021. (**a**)—spring; (**b**)—summer; (**c**)—autumn; (**d**)—winter. Points indicate the location of sampling stations.

Seasonal SST, SSS, and Chl-a maps were created by averaging each pixel of respective cruise maps during the whole study period (Figure 4, Figure 5 and Figure 6). Spatial distributions of SST, SS, and Chl-a demonstrated seasonal variations. In spring, SST ranged from −1.5 °C to 6.9 °C with maximum values in May. The southern and south-western regions had the warmest SST (Figure 4a). 

In summer, SST was in a range of −0.8–+10.0 °C with maximum values in the coastal regions of the southern and south-eastern areas (Figure 4b). In autumn, SST varied between −1.7 °C and 10.9 °C, with the highest value in the south-eastern Barents Sea (Figure 4c). In winter, SST ranged from −1.8 to 7.2 °C, decreasing from the south to the north. Minimum values near zero or just below were typical for most northern areas in all seasons (Figure 4). SSS varied between 8.35 psu and 35.10 psu, and the average values were in a range of 22.0–35.0 psu (Figure 5). SSS patterns were similar in all seasons, with the highest estimations in the central and western regions and minimal SSS in the south-eastern Barents Sea (Figure 5). 

Clear spatial patterns were found for Chl-a (Figure 6). In spring, Chl-a concentrations were 0.01–8.25 mg m^–3^, with the highest measures in the north-western, northern, and eastern regions (Figure 6a). In summer, Chl-a content varied from 0.03 to 12.36 mg m^–3^, with the maxima of Chl-a being located in the western, central, and southern regions (Figure 6b). In autumn, Chl-a concentrations ranged between 0.08 and 1.48 mg m^–3^ with an average of 0.55 mg m^–3^, with the highest values recorded in the western and south-eastern areas (Figure 6c). In winter, Chl-a varied from 0.05 to 0.28 mg m^–3^, with the highest records in Svalbard coastal waters and the central region (Figure 6d). Multiple comparisons of the mean SST, SSS, and Chl-a showed significant seasonal differences in the case of SST (Table 2). The highest SSTs were found for the summer–autumn period. Salinity did not demonstrate any seasonal pattern (Tukey’s pairwise comparisons, *p* > 0.05) (Table 2). The mean values of Chl-a were similar in spring, summer, and autumn, but they differed significantly from the concentrations obtained in winter (Table 2).

**Figure 7:**
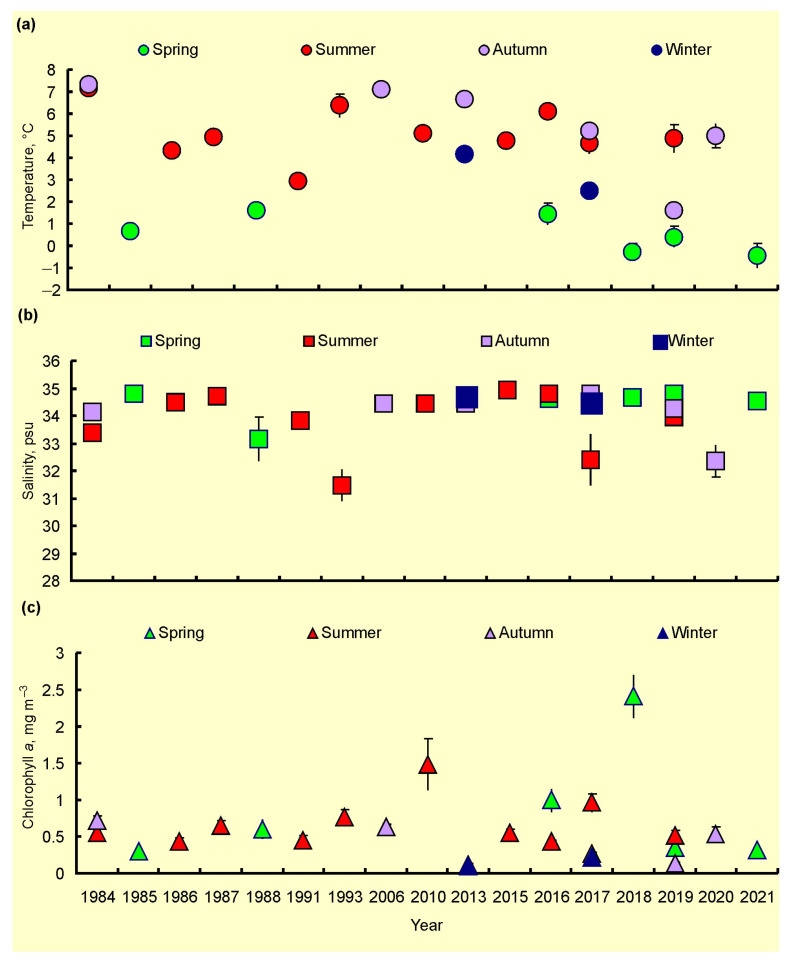
Temporal variation in SST, °C (**a**), SSS, psu (**b**), and Chl-a, mg m^–3^ (**c**) in the Barents Sea, 1984–2021.

Temporal variations in the mean values of SST, SSS, and Chl-a are presented in Figure 7. SST tended to decrease from 1984 to 1988 and then increase till 2016 in the summer periods (Figure 7a). The autumn and spring estimations demonstrated a general declining trend from 1984 to the 2010s (Figure 7a). The mean SSSs were rather stable over the study period (Figure 7b). Chl-a showed an increasing trend for all seasons from the 1980s to 2010s (Figure 7c). 

**Figure 8:**
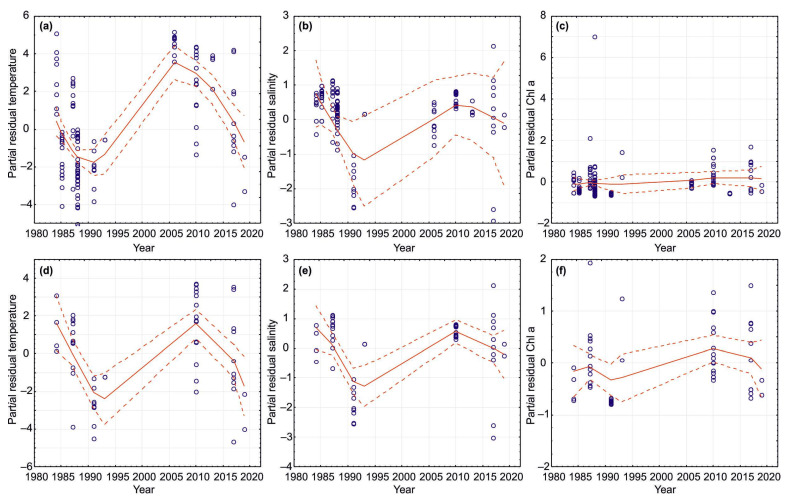
GAM results (parametric, normal added functions) for SST, SSS, and Chl-a as response variables in relation to year as explanatory variable at the Kola Section (Barents Sea, 69°30′–74°30′ N, 33°30′ N) during the period 1984–2021. (**a**–**c**)—all data combined; (**d**–**f**)—summer data.

Analysis of summer and combined data for the Kola Section indicated that there was significant temporal variability in SST, SSS, and Chl-a during the study period (Table 3 and Table 4, Figure 8). Linear regression analysis revealed a significant positive trend in SST (combined data) (Table 3). SST and Chl-a demonstrated a slight increase during the study period (Table 3). GAMs showed that SST was positively related to year (Figure 8), which explained more than 43% of the temporal variability in SST (Table 4). Summer SSS increased from 1984 to 2021 (Figure 8), while combined SSS demonstrated no clear pattern (Table 4). Chl-a showed a slight increase over the study period (Figure 8). Year (time) as a predictor explained <17–27% of temporal variability in Chl-a in the summer season, while in all seasons, it explained <3–5% of the total Chl-a variability (Table 4).

### 3.3. The Impact of Climatic Factors on Chl-a

**Figure 9:**
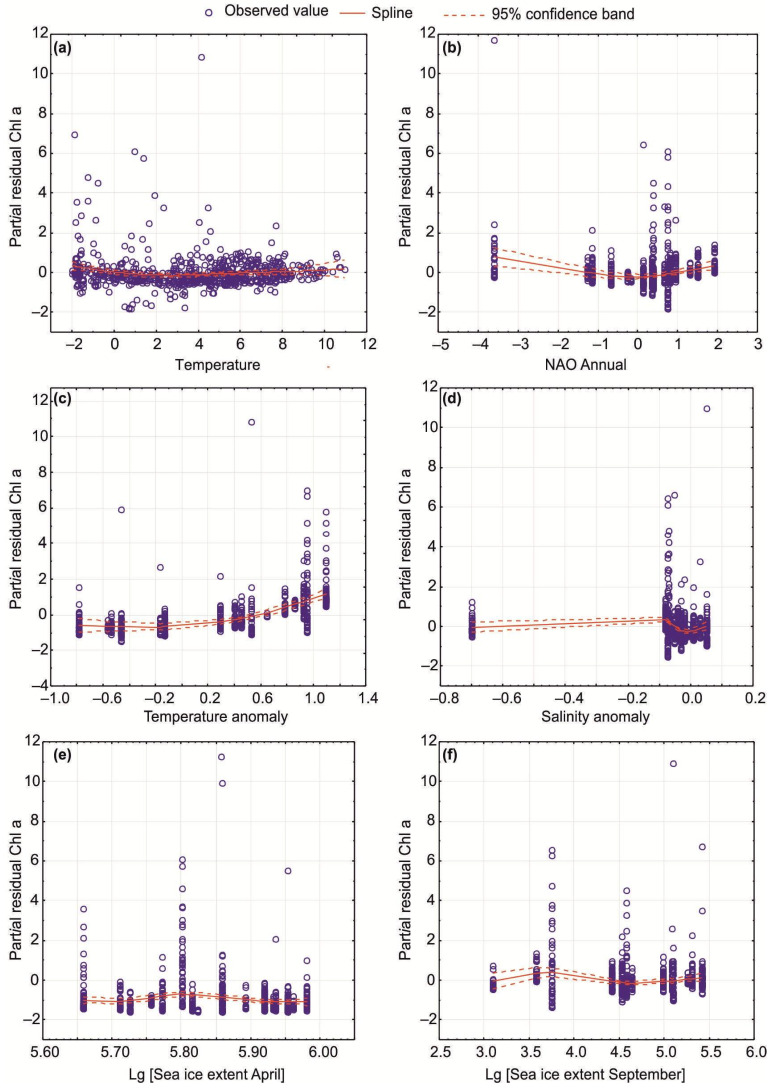
Significant GAM results (parametric, normal added functions) for Chl-a as a response variable in relation to climatic predictors as explanatory variables in the Barents Sea during the period 1984–2021. Smoothed best-fit lines with 95% confidence intervals for combined data (all seasons) are presented. Climatic predictors: (**a**)—SST; (**b**)—annual NAO index; (**c**)—temperature anomaly at the Kola Section; (**d**)—salinity anomaly at the Kola Section; (**e**)—sea ice extent in April; (**f**)—sea ice extent in September.

Temporal patterns for Chl-a in relation to SST, SSS, and selected climatic indices were evaluated using GAMs. Parametric and non-parametric GAMs revealed that environmental variables explained 25–34% of the total fluctuations in Chl-a in all seasons (Table 5). Normal-based GAMs showed significant trends for Chl-a during the study periods, with six predictors being the most important: SST, annual NAO index, temperature and salinity anomalies at the Kola Section, and sea ice extent in April and September (Figure 9, Table 5 and Table 6). The climatic dataset best explained variations in Chl-a in the spring and autumn seasons (Table 5). The GAMs indicated higher Chl-a values at increasing temperatures (>4–6 °C) and positive NAO indices (Figure 9, Table 6). Enhanced Chl-a was predicted when temperature anomalies at the Kola Section would be >0 (Figure 9, Table 6). An opposite pattern was found for salinity anomalies (Figure 9, Table 6). Lower Chl-a was associated with greater ice extent in September and April, as well as annual sea ice coverage (Figure 9, Table 6). Poisson-based GAMs estimated similar results suggesting adequate predictions for our dataset (Table 5 and Table 6). Significant seasonal GAMs were obtained only in the case of the spring period when salinity anomaly and annual sea ice coverage were considered (Figure 9, Table 6).

## 4. Discussion

The present study provided an analysis of long-term variations in water temperature, salinity, and Chl-a measured in situ in the surface layer of the Barents Sea. Important limitations of our work must be mentioned. Firstly, our dataset has some temporal gaps for SST, SS, and Chl-a. Secondly, some regions were not fully investigated, especially the northern and north-western areas (spatial gaps in data). Thirdly, we had a restricted dataset for the winter season (only two periods) that was associated with severe environmental conditions preventing successful sampling. However, we were able to investigate spatial and temporal patterns of SST, SSS, and Chl-a in the Barents Sea and adjacent waters using interpolation procedures and GAMs. In general, we confirmed our hypothesis that large-scale atmospheric phenomena, climatic indices (anomalies of temperature and salinity in a secular oceanographic transect), and sea ice extent fluctuations in the region were responsible for temporal variations in Chl-a.

### 4.1. Temporal Variations in Climatic Indicators

Clear climatic variations have been documented during the past decades in the northern hemisphere [47,54,55,56]. The Nordic Seas and the Arctic Ocean have been warming since the end of 20 century [6,7,12,54]. Global atmospheric processes, which can be expressed by using some indices (e.g., NAO and the Arctic Oscillation index), were found to be responsible for climatic changes in the Arctic region, including the Barents Sea and adjacent waters [14,57]. Analysis of NAO variations from 1984 to 2021 suggests a generally positive trend since the 2010s. NAO strongly affects large-scale atmospheric variations in the Arctic Ocean. In particular, NAO significantly affects the magnitude of the flow of warm AW from the Norwegian Sea to the Barents Sea [14]. A positive winter NAO index is usually associated with the prevalence of southwesterly winds that are responsible for the enhanced inflow of AW into the Arctic Ocean through the Barents Sea [57]. Therefore, the years with positive NAO may be characterized as warm periods. Other oceanographic effects of the positive NAO mode include lower Polar surface air pressure, a high degree of ice melting in the Barents Sea and Arctic Ocean, eastward shifting of the Polar Front, more northerly storm tracks, higher air temperatures, and increased heat transport to the northern regions [14,55]. Another important factor influencing AW inflow in the Barents Sea is the wind fields between Bear Island and Norway [14]. Climatic conditions of the Barents Sea vary from cold to warm phase [11,19], and our study period included mainly warm periods.

Analysis of published data regarding variations in the water temperature and salinity anomalies at the Kola Section [12,15,16,49] during 1984–2021 revealed a longer duration of warm phases relative to moderate and cold periods. We also noted a general trend for the temperature anomaly to increase over the study period. Considering that the anomalies were calculated for the stations located in AW, a warming tendency has been encountered from the beginning of the 21 century in accordance with general warming in the Arctic Ocean [3,4,6,7]. Simultaneous changes in the ice cover have been observed in the Arctic Ocean and Barents Sea during the study period [4,7,17,58]. A significant decline in the maximum (April) and annual ice coverage has been evident since 2000 [51]. However, this trend was not so obvious in recent years, with a general decrease in the ice extent from 1984 to 2021 [15,16]. Thus, climatic conditions in our study fluctuated in a wide range, with a tendency for the water temperature to be higher and for the ice extent to be lower than usual.

### 4.2. Spatial, Seasonal, and Temporal Variations in SST, SSS, and Chl-a

The spatial distributions of SST, SSS, and Chl-a in the Barents Sea demonstrated obvious variability in each sampling season. SST was found to be highest in the western and southern regions during the spring period, and this pattern reflected the influence of warm AW flowing from the Norwegian Sea. Minimal SST was encountered in the northern parts of the sea, where cold currents prevailed. In the summer and autumn seasons, the highest SST was also recorded in CW, especially in the south-eastern and southern Barents Sea. These observations can be explained by seasonal heating and shallowness of the inshore waters [11,19]. The average SST was similar during the summer-autumn periods while differed significantly from the SST in spring and winter. This result is associated with the seasonal sunlight regime in the Arctic, where the lowest temperatures occur during the polar night in winter. Lower spring temperatures can be explained by the winter cooling of waters [13,19]. 

SSS showed similar spatial distributions as SSTs, with the highest seasonal values in the western and central regions where high-saline AW was present. The northern regions affected by less saline Artic waters had lower salinity partly due to ice melting. Coastal regions with extensive river run-off also demonstrated lower salinity, and this is a common pattern typical for freshwater-affected areas in the Nordic Seas [2,14]. Despite pronounced spatial variations between various regions, the average SSSs were similar in all seasons in accordance with multi-year observations in the Barents Sea during 1950–2000 [49]. 

First peaks of Chl-a were detected in the spring period in the northern and central parts of the Barents Sea, suggesting earlier phytoplankton bloom in ArW compared to AW, where the outbursts of microalgae were 1–1.5 months later. Our spring averaged estimations were comparable with the results of previous findings that recorded maximum Chl-a values in the Marginal Ice zone of the Barents Sea [59,60,61]. Therefore, the ice edge and frontal zones may be considered the most productive environments in the Arctic during the spring time. We also noted high Chl-a concentrations in the eastern regions that agree with the results obtained by Dalpadado et al. [39], who reported the north- and eastward expansion of the satellite-derived Chl-a distribution associated with earlier spring blooms and higher concentrations in the eastern regions during warm periods. Phytoplankton peaks in AW and CW are usually registered later in the season, leading to higher Chl-a values in the summer season. The influence of more regular replenishment of nutrients to the euphotic layer is certainly one of the main factors responsible for the common observation that the production of phytoplankton in inshore waters is usually considerably greater than in the open sea [62]. The decline in phytoplankton density in AW and CW after the spring maximum occurs during June–July due to rapid nutrient depletion [19,40]. In contrast, during June and July, areas of spring phytoplankton outbursts in ArW were found in the northern and north-eastern regions, and these were associated with the retreating ice edge [60,61]. 

Our study provided new data regarding the Chl-a distribution in less-studied and hard-to-access regions of the Barents Sea. We revealed high concentrations of Chl-a in the north-eastern and northern regions during the spring and summer seasons along the ice melting edge. Our study documented a relatively high density of Chl-a near Novaya Zemlya (eastern Barents Sea), where intense blooms were revealed in frontal zones and cold ArW. In autumn, the maxima of Chl-a were located mainly in CW of the Barents Sea and in the open regions in the west. Autumn bloom is characterized by lower Chl-a values relative to spring estimates. The duration of autumn peaks is also shorter [14,40], and our study confirmed this general observation. 

We found higher Chl-a density in the south-eastern Barents Sea in the summer and autumn periods. These regions are strongly affected by freshwater run-off from the Pechora River. The run-off from the land to sea areas may carry sufficient amounts of nutrient salts to stimulate phytoplankton blooms in the inshore regions [61]. Arctic rivers carry considerable quantities of nitrate and phosphate as well as other essential minerals [14], and these may be responsible for the maintenance of relatively high levels of Chl-a in inshore waters. Previous studies have also established that phytoplankton abundance and biomass were higher in the Pechora Sea than in the open sea [14,40]. 

During winter, phytoplankton production is low, and the distribution of nutrients in the water column is more homogeneous. Studies focused on the winter Chl-a are scarce, and our investigation provides new insights regarding Chl-a patterns in the Barents Sea. Although our winter dataset was limited, we were able to create a map showing the Chl-a pattern in the western part of the sea. High concentrations of Chl-a were revealed in AW, suggesting a positive influence of the warm inflow on the phytoplankton. The second site where high Chl-a was found in Svalbard coastal waters. The western area near Svalbard represents a region with dominating warm AW that might be favorable for phytoplankton growth. The waters east of Svalbard are a frontal zone with interacting AW and ArW [11,14] that can explain the high Chl-a density in this region.

Our study revealed a general trend for SST to increase from the end of 20 century. This observation is in line with the global warming processes documented over the past decades [3,6,7,12]. However, our spring and autumn measures demonstrated a slight tendency to decrease. We may speculate that a lack of spatial and temporal data and/or the irregular location of sampling stations are reasons for this pattern. Another possible explanation is the difference in sampling dates. During the 1980s, the main sampling was conducted in the late spring and early autumn, while during the 2010s, we recorded SST mainly in early spring and late autumn when the water temperatures were lower. However, analysis of data for the Kola Section indicated an increase in water temperature in spring and summer, confirming the warming trend in the Barents Sea.

SSS showed no temporal variations during the study period, although in 2015 and 2016, SSS values were maximal. These high estimates indicate a good correspondence with thermal conditions. The period 2015–2016 was one of the warmest periods in the 21 century in the Barents Sea. Considering a strong association of the surface water heating with the degree of AW inflow [11,12,14,16], the greater SSS can be explained by the stronger influence of AW advected from the Norwegian Sea. 

We found that Chl-a tended to increase during the study period. Plankton communities are very sensitive to environmental forcing and exhibit different responses to climatic influence [20,22,23,25]. Planktonic microalgae can react to climatic changes, and this may be encountered through integral parameters such as primary production, total annual stock, phytoplankton abundance, and biomass [10,31]. Chl-a is a good indicator of phytoplankton density in the Arctic and, therefore, may be used to predict environmental responses of pelagic microalgae to environmental perturbations [35,36,39]. Our data suggest an overall positive response of the phytoplankton to warming noted in the Arctic and, in particular, in the Barents Sea. For instance, Lewis et al. [63] observed an increase in Chl-a in the Arctic Ocean and in the Barents Sea by 21.5% and 60.5%, respectively, during 1998–2018. Moreover, other recent studies have shown that satellite-derived values SST, Chl-a, and primary production have increased during the last two decades. In particular, in the whole Barents Sea, satellite-based new primary production doubled during the 20-year period from 1998 to 2017, which is equivalent to an annual 2.9 Tg C increase [39]. Thus, recent environmental changes appear to be responsible for the enhanced Chl-a estimations observed since the 2000s.

### 4.3. Environmental Impact of Climatic Indicators on SST, SSS, and Chl-a

GAMs obtained in this study confirmed our hypothesis regarding the environmental impacts of the climatic factors on the inter-annual variability of in situ Chl-a in the surface layer. We revealed that a considerable part of Chl-a variations could be explained by SST, SSS, NAO indices, water temperature/salinity anomalies, and ice conditions in the Barents Sea. However, only six predictors were found to contribute significantly to the Chl-a dynamics. The GAMs predicted enhanced Chl-a levels during the periods with positive NAO, increased SST, positive temperature anomalies at the Kola Section, and decreased sea ice extent in April or September. Many previous studies have shown strong correlations between NAO indices and water temperature in the Arctic [57,64,65,66]. Increasing water temperature results in ice loss in the marginal seas of the Arctic Ocean and might strongly influence phenology and seasonal phytoplankton stocks in the Barents Sea. Similar to our observation, McGinty et al. [67] revealed a positive correlation between satellite-derived and in situ Chl-a values and SST in the waters near Iceland.

Positive NAO phases noted in our study led to an increase in AW inflow, enhanced heating of the surface waters, and earlier ice melting, resulting in better conditions of more area of the open water for phytoplankton growth and, thus, greater Chl-a values. The temperature was found to have a positive effect on phytoplankton production in the Arctic, especially during the early bloom in the spring [68,69]. However, the major effect of increasing water temperature is connected to the stabilization of the water column. The stability of a water column depends, to a large extent, on the temperature conditions, so slight warming of the surface layers would cause them to become less dense, restricting vertical mixing with the underlying layers and causing the stability of the water column [62]. It is probable that low ice cover might enhance nutrient concentrations due to vertical mixing. Warmer surface temperatures have been found to lead to a clear pycnocline preventing the transport of nutrients from deeper layers into the upper euphotic zone. However, in particular regions of the Barents Sea (banks and submarine plateaus), there may be strong vertical mixing to provide high nutrients at the surface, even during summer periods. However, such conditions may be encountered occasionally, and these are caused by certain winds and storms [62,63]. Therefore, positive NAO periods may be associated with an earlier formation of the stability in the euphotic layer that allows earlier spring outbursts and can lead to higher primary production and Chl-a, at least in ArW of the Barents Sea. 

The summer-time stratification in the northern part of the Barents Sea may also increase as a result of greater ice melting and glacial discharge near Franz Joseph Land, Svalbard, and Novaya Zemlya. Slight changes in salinity due to increased inflow of high-saline AW during the periods with positive NAO may also play a role in the stabilization of the water column causing earlier spring outbursts in the Barents Sea during the spring and summer periods. In the northern and central Barents Sea, the major diatom peak does not appear to begin with the return of sufficient daylight, and it appears to follow rapidly upon the ice melting in the Marginal Ice Zone [40,41,60]. Successful flowering of the microalgae needs some stabilization of the water column so that the diatoms are not carried by turbulence out of the euphotic layer. Earlier phytoplankton blooms in the CW during the summer periods in the present study in the 2010s can be partly explained by lower salinity due to earlier ice melting and increased freshwater run-off that play a significant role in forming stability of the upper layers. 

There is evidence that positive NAO phases are related to a higher degree of AW advection in the Barents Sea [6,14]. The advective influx might play a role in determining plankton assemblages in the regions studied. For instance, the increased Chl-a measures recorded in our study in warm periods could also be attributed to more rich phytoplankton assemblages transported with AW from the Norwegian Sea [11,23,25]. Another explanation is a possible upwelling process occurring along the shelf break in the Arctic Ocean when certain conditions are met. This situation has been recorded in the northern Svalbard waters, where a northward retreat of the ice edge was present, together with favorable along-shelf winds, leading to increased offshore Ekman transport and resulting in higher primary production [70]. Similar highly productive Chl-a areas might be formed in other regions of the Barents Sea with ice-free areas and zones of low atmospheric pressure. In the Arctic Ocean, greater Chl-a contents have been reported along the interior shelf break where upwelling events resulted in the movement of nutrient-rich water from the deep basin towards the nutrient-depleted upper euphotic layer [63,69].

Our study suggests negative correlations between the extent of sea ice and Chl-a in the Barents Sea. Ice conditions strongly affect the distribution of Chl-a in the Arctic Ocean and adjacent waters, and this influence is mainly connected with light conditions. The spring bloom is strongly dependent on the retreating of the sea ice that determines the amount of solar radiation in the upper euphotic layer zone [4,9,71]. In contrast, nutrient replenishment in the euphotic zone can be considered one of the main factors driving phytoplankton bloom in summer due to sufficient light availability [9]. Therefore, the seasonal stratification of the upper zone can be reduced by the positive anomaly of the sea ice, and this may lead to changes in Chl-a distributions.

Moreover, ice extent anomalies were found to have direct consequences for the spatial distribution of spring blooms in the Barents Sea [72]. In years with minimal sea ice extent, two spatially distinct blooms were observed (along the ice edge and in ice-free water), and these blooms were triggered by different stratification patterns: heating of the surface layers in ice-free water and ice melting near the ice edge [72]. A previous study also found northward and eastward shifts in the spring and summer phytoplankton blooms in the Barents Sea during the period 1998–2014 [72]. It has been emphasized that the melting of sea ice creates a stable shallow mixed layer providing optimal light and nutrient conditions and preventing phytoplankton from vertical excursions out of the euphotic layer [72]. A multi-year study compared Chl-a in the Greenland and Barents Seas revealed clear spatial trends strongly related to ice conditions [73] with seasonal peaks in April or May. Earlier and higher ice melting strongly affected Chl-a blooms in the Barents Sea [73]. Dong et al. [74] have pointed out that SST and sea ice conditions had greater importance for phytoplankton dynamics in the northern Barents Sea compared to the southern regions. They revealed an earlier spring phytoplankton bloom and a higher magnitude of satellite Chl-a estimations in warm years over the period 1998–2014 [74]. Similar to our observations, there was a non-linear association between the timing of the sea-ice retreat and the phytoplankton peak, with spring bloom occurring before or immediately following the ice retreat [73]. Therefore, more extensive ice melting in the periods with positive NAO may be thought as the main reason for enhanced Chl-a and earlier blooms in the Arctic seas.

Changes in ice cover recorded in the Arctic during the past decades may be a significant factor affecting ice algae. In the Barents Sea, the total ice algal production is an important source of primary production in the northern ice-covered regions and the Marginal Ice Zone. However, it has been found that under-ice bloom supports only 6% of the total annual primary production in that area [19]. Although we did not measure under-ice algal Chl-a, we must notice that the interannual variability in the total Chl-a recorded in our study might be partly associated with changes in the abundance of sea-ice algae. We can expect a decline in the total under-ice algal biomass in warmer years due to a retreat of ice cover northward. Therefore, we think that the overall contribution of ice-related microalgae to the total fluctuation of Chl-a in the Barents Sea would be less-significant compared to other factors, especially in the periods with positive NAO.

We also must emphasize that the models obtained in our study explained only part of Chl-a variability in the Barents Sea, suggesting that other drivers besides climatic factors would be important in determining phytoplankton dynamics in the Barents Sea. Phytoplankton growth and development are controlled by a set of environmental drivers, with light intensity, nutrient availability, and grazing impacts being the most significant [40,61,62]. 

Light conditions in the Arctic demonstrate a clear seasonal pattern from the continuous daylight in summer to the periods of darkness during the polar night in winter [11,14]. Changes in the ice cover may be responsible for fluctuations in the extent of the open sea, depth of the light penetration, and duration of the productive season in the Barents Sea. Light conditions in the Arctic seas also depend on cyclonic activity and cloudiness. For instance, cyclones have been found to be important drivers forcing the winter sea-ice extent in the Barents Sea. More intense cyclones caused higher sea-ice cover in the Barents Sea through the advection of sea ice from the Arctic Ocean by the cyclone-associated winds [75]. An anomalous high pressure over the Arctic Ocean can lead to a decrease in cloudiness in the upper and middle levels of the atmosphere, possibly associated with decreased storm activity in the marginal seas, including the Barents Sea [76]. There is evidence that the dominance of low clouds in the Arctic is associated with the increase in downward longwave radiation [76]. Photosynthetically active radiation (PAR) and primary production (PP) have been detected to be controlled by increasing cloudiness during summer in the Arctic Ocean. Although there was a decrease in the PAR and PP, Chl-a tended to increase due to sea-ice loss in perennially and seasonally open waters [77].

The importance of nutrient supply in phytoplankton growth and primary production has been widely recognized in many studies [25,40,60,61,62]. It is known that AW in the Barents Sea is a major source of nutrients flowing into the Barents Sea [11,14]. Recent studies have reported an increased influx of AW into the Barents Sea [15,16]. The proportion of AW relative to ArW has increased during the 2010–2020s, suggesting higher nutrient stocks available for phytoplankton development. Waters of Atlantic origin have been found to be the most nutrient-rich among all types of water masses in the Barents Sea [14]. Considering a strong association between AW inflow and NAO, we can predict higher levels of Chl-a in the Barents Sea during phases with positive NAO, and this increase may be partly associated with the higher nutrient concentrations. 

Many studies have pointed out the significance of the trophic relationship between phytoplankton and zooplankton in the Arctic ecosystems. The high contribution of zooplankton grazing to the decline of phytoplankton stock and the role of zooplankton ingestion in controlling the Arctic marine food web structure is more evident during the periods of spring and summer blooms. Positive correlations between Chl-a and zooplankton abundance have been reported previously in the Barents Sea [78,79,80]. Zooplankton biomass is an indicator of climatic changes, and it can increase in warm years owing to favorable temperatures and greater food availability [25,26,27,39,81,82,83,84].

Therefore, the amount of light penetrating the surface, nutrient concentrations, and zooplankton grazing together with climatic factors must be included in the improved models in order to better predict temporal and spatial trends in Chl-a in the Arctic Ocean and adjacent marginal seas.

## 5. Conclusions

Model approaches are a powerful tool for investigating relations between biological systems and environmental fluctuations. Our study showed a good correspondence between measured variables in situ (surface sea temperature, salinity, and chlorophyll *a* levels) and a set of environmental factors, including the North Atlantic Oscillation indices, temperature/salinity anomalies at the Kola Section, and sea ice extent in the Barents Sea. Positive temperature anomalies and a decreasing ice extent have been noted since the 2000s. There were significant spatial and temporal trends in SST, SSS, and Chl-a over the period of 1984–2021. SST and Chl-a tended to increase from the maximum values noted in 2015 and 2016. Spring and summer values of Chl-a were found to be higher compared to the autumn and winter estimations. Our study documented relatively high concentrations of Chl-a in the Marginal Ice zone and near the retreating ice edge in the spring seasons, while maximal estimates of Chl-a were encountered in the coastal regions during the summer seasons. Generalized additive models predicted enhanced values of Chl-a in warm periods. Considering continuous climatic changes in the Arctic associated with the heating of water masses and reducing ice coverage, we may propose a subsequent increase in the total phytoplankton stock and Chl-a in the Barents Sea. Rising phytoplankton productivity may have various effects, such as altering pelagic communities, fluctuations in fish and shellfish stocks, and changes in marine biodiversity. Our study provides a basis for future investigations focusing on the ecosystem fluctuations in the Arctic Ocean and adjacent regions associated with climate forcing. For a better understanding of the ecosystem dynamics and productivity in the Barents Sea, further studies with better space-time resolution are needed.

## Figures and Tables

**Table 1:** List of cruises for Chl-a in the Barents Sea, 1984–2021.

Cruise	Period	Researh Vessel	Number of Samples
1	August–September 1984	*Pomor*	46
2	August–September 1984	*Dalnie Zelentsy*	29
3	August 1984	*Akhill*	24
4	April 1985	*Pomor*	29
5	June 1986	*Dalnie Zelentsy*	17
6	June–July 1987	*Dalnie Zelentsy*	48
7	March–May1988	*Dalnie Zelentsy*	28
8	March–May1988	Coastal station	28
9	June–July 1991	*Dalnie Zelentsy*	61
10	June 1993	*Dalnie Zelentsy*	33
11	June 1993	*Dalnie Zelentsy*	16
12	July 1993	*Dalnie Zelentsy*	23
13	September–October 2006	*Dalnie Zelentsy*	18
14	August 2010	*Dalnie Zelentsy*	34
15	November 2013	*Dalnie Zelentsy*	10
16	June–July 2015	*Dalnie Zelentsy*	52
17	April–May 2016	*Dalnie Zelentsy*	51
18	June–July 2016	*Dalnie Zelentsy*	52
19	July 2017	*Dalnie Zelentsy*	30
20	November–December 2017	*Dalnie Zelentsy*	27
21	April–May 2018	*Dalnie Zelentsy*	46
22	April 2019	*Dalnie Zelentsy*	30
23	June 2019	*Dalnie Zelentsy*	12
24	November 2019	*Dalnie Zelentsy*	6
25	September 2020	*Dalnie Zelentsy*	6
26	October 2020	*Dalnie Zelentsy*	9
27	March–April 2021	*Dalnie Zelentsy*	38
	Total		803

**Table 2:** Mean SST (°C), SSS (psu), and Chl-a (mg m^–3^) in the Barents Sea, 1984–2021. Letters show significant differences between seasons (Tukey’s pairwise comparisons, *p* < 0.05).

Parameter	Season			
	Spring (A)	Summer (B)	Autumn (C)	Winter (D)
Temperature				
min–max	−1.7–10.9	−2–+7	−0.8–+12.2	−1.8–+7.3
mean ± SE	0.7 ± 0.1 ^BCD^	5.2 ± 0.1 ^AD^	6.2 ± 0.3 ^AD^	3.0 ± 0.6 ^ABC^
Salinity				
min–max	27.55–35.04	8.25–35.3	6.16–35.22	34.07–35.07
mean ± SE	34.32 ± 0.18	33.85 ± 0.11	34.01 ± 0.13	34.55 ± 0.07
Chlorophyll *a*				
min–max	0.08–1.65	0.01–8.25	0.03–12.36	0.05–0.28
mean ± SE	0.9 ± 0.09 ^D^	0.66 ± 0.04 ^D^	0.55 ± 0.04	0.18 ± 0.02 ^AB^

**Table 3:** Results of linear regression analyses related to SST, SSS, and Chl-a at the Kola Section (Barents Sea, 69°30′–74°30′ N, 33°30′ N) during the period 1984–2021 with year as an independent variable.

Season	Slope	Intercept	F	*p*	R^2^
SST					
Summer	0.022 ± 0.025	–39 ± 50	0.75	0.390	0.014
All	0.106 ± 0.018	–209 ± 37	33.87	<0.001	0.208
SSS					
Summer	0.013 ± 0.012	8 ± 25	1.10	0.230	0.020
All	0.027 ± 0.030	–19 ± 60	0.76	0.385	0.006
Chl-a					
Summer	0.012 ± 0.007	–23 ± 14	2.89	0.095	0.053
All	0.010 ± 0.006	–19 ± 12	2.56	0.112	0.019

**Table 4:** GAM parameters showing temporal variations of SST, SSS, and Chl-a as response variables in relation to year as explanatory variable at the Kola Section in the Barents Sea during the period 1984–2021 using parametric (Normal) and non-parametric (Poisson) added functions.

Season		GAM Coefficient (β)	Standard Error	Standard Score	R Square × 100%	Non-Linear *p*-Value
		SST				
Summer	Normal	0.022	0.019	1.11	43.6	<0.001
	Poisson	0.056	0.022	2.57	45.3	<0.001
All	Normal	0.106	0.014	7.33	52.7	<0.001
	Poisson	0.103	0.016	6.24	49.8	<0.001
		SSS				
Summer	Normal	0.013	0.010	1.30	40.4	<0.001
	Poisson	0.017	0.000	42.95	40.1	0.935
All	Normal	0.006	0.018	0.33	4.9	0.178
	Poisson	0.006	0.044	0.14	3.6	0.897
		Chl-a				
Summer	Normal	0.012	0.007	1.77	17.4	0.124
	Poisson	0.015	0.009	1.65	27.8	0.144
All	Normal	0.009	0.006	1.52	3.0	0.810
	Poisson	0.009	0.007	1.42	5.5	0.773

**Table 5:** Summary statistics for GAM to predict Chl-a patterns in relation to selected climatic predictors (SST, SSS, Annual NAO index, winter NAO index, annual water temperature and salinity anomalies, annual/April/September ice coverage) in the Barents Sea during 1984–2021 using parametric (Normal) and non-parametric (Poisson) added functions.

Added Function	Final Deviance	Residual Df	Number of Observations	Outer Iteration Number	Number of Smooths	Scale Estimate	R Square × 100%
All seasons							
Normal	547.9	716.0	753	1	15	0.765	25.8
Poisson	365.4	715.4	753	18	243	1.000	34.5
Spring							
Normal	296.8	212.0	249	1	15	1.400	35.4
Poisson	165.0	211.5	249	20	300	1.000	47.2
Summer							
Normal	200.4	358.0	395	1	15	0.560	19.0
Poisson	126.2	357.4	395	20	300	1.000	35.0
Autumn							
Normal	6.4	55.8	92	1	15	0.115	48.4
Poisson	9.3	55.7	92	20	300	1.000	57.3

**Table 6:** GAM assessed Chl-a patterns in relation to selected climatic predictors in the Barents Sea during the period of 1984–2021 using parametric (Normal) and non-parametric (Poisson) added functions.

	Normal				Poisson			
Climatic Predictor	GAM Coefficient (β)	Standard Error	Standard Score	Non-Linear *p*-Value	GAM Coefficient (β)	Standard Error	Standard Score	Non-Linear *p*-Value
All seasons								
SST	0.012	0.012	−1.018	<0.05	0.010	0.011	0.925	0.033
SSS	0.024	0.016	1.522	0.838	0.020	0.008	2.578	0.474
NAOAn	−0.052	0.056	−0.916	<0.001	0.034	0.045	0.762	<0.001
NAOW	0.056	0.057	0.984	0.911	−0.027	0.047	−0.586	0.646
dT	1.020	0.156	6.546	<0.001	1.253	0.140	8.922	<0.001
dS	−0.130	0.204	−0.637	<0.001	−0.528	0.149	−3.544	<0.001
Annual SIE	−0.665	0.230	−2.880	<0.001	−0.564	0.197	−2.862	<0.001
April SIE	−0.380	0.336	0.926	<0.001	−0.318	0.270	−1.170	0.783
September SIE	−0.198	0.059	−3.337	<0.001	−0.200	0.073	−2.749	<0.001
Spring								
SST	−0.065	0.038	−1.708	0.502	−0.042	0.023	−1.809	0.064
SSS	0.015	0.027	0.560	0.080	0.017	0.009	1.965	0.371
NAOAn	−0.210	0.346	−0.608	<0.05	−2.668	0.349	−7.643	0.669
NAOW	−0.100	0.307	−0.326	0.926	−2.239	0.297	−7.537	0.990
dT	0.894	1.564	0.572	0.231	11.240	1.749	6.428	0.106
dS	0.978	0.738	1.326	<0.001	4.758	0.751	6.337	<0.001
Annual SIE	−1.933	0.437	−4.417	<0.05	−1.343	0.323	−4.153	<0.05
April SIE	−0.639	1.358	−0.471	0.882	−9.081	1.488	−6.101	0.868
September SIE	0.000	0.000		0.884	0.000	0.000		0.996
Summer								
SST	0.070	0.019	3.636	0.265	0.072	0.017	4.155	0.640
SSS	0.057	0.029	2.002	0.829	0.068	0.026	2.625	0.581
NAOAn	−0.045	0.065	−0.695	0.988	−0.033	0.058	−0.574	0.999
NAOW	−0.021	0.070	−0.296	0.991	−0.058	0.065	−0.896	0.998
dT	0.584	0.223	2.618	0.881	1.022	0.236	4.322	0.952
dS	0.880	0.324	2.717	<0.05	0.448	0.305	1.469	<0.001
Annual SIE	−0.039	1.150	−0.034	0.650	1.022	1.038	0.984	0.999
April SIE	3.359	1.718	1.955	0.794	4.635	1.739	2.666	1.000
September SIE	−0.212	0.233	−0.911	0.999	−0.532	0.218	−2.434	0.968
Autumn								
SST	0.054	0.021	2.540	0.435	0.036	0.037	0.975	0.913
SSS	−0.003	0.043	−0.063	0.717	−0.019	0.089	−0.210	0.993
NAOAn	0.200	0.399	0.503	0.239	−0.049	0.641	−0.077	1.000
NAOW	0.017	0.258	0.065	0.979	0.056	0.367	0.152	1.000
dT	0.129	0.159	0.810	0.812	−0.428	0.296	−1.445	0.988
dS	−0.719	0.682	−1.054	0.194	−0.057	1.142	−0.050	1.000
Annual SIE	−1.008	1.116	−0.904	0.815	−2.769	1.862	−1.487	1.000
April SIE	0.249	0.326	0.764	0.718	0.820	0.616	1.332	1.000
September SIE	0.000	0.000		0.958	0.000	0.000		0.995

## Data Availability

The data are available on request, from the corresponding author.

## Supplementary Materials

The following are available online at https://www.mdpi.com/article/10.3390/biology12010119/s1, Figure S1: Histograms of data distributions (surface chlorophyll *a*, water temperature and salinity) in the Barents Sea in 1983–2021. Normality was tested with the Shapiro–Wilk test at α = 0.05.

## References

[1] Field C.B., Behrenfeld M.J., Randerson J.T., Falkowski P. (1998). Primary production of the biosphere: Integrating terrestrial and oceanic components. Science.

[2] Longhurst A. (2007). Ecological Geography of the Sea.

[3] ACIA (2005). Arctic Climate Impact Assessment.

[4] Meier W.N., Hovelsrud G.K., van Oort B.E.H., Key J.R., Kovacs K.M., Michel C., Haas C., Granskog M.A., Gerland S., Perovich D.K. (2014). Arctic sea ice in transformation: A review of recent observed changes and impacts on biology and human activity. Rev. Geophys..

[5] Carmack E., Polyakov I., Padman L., Fer I., Hunke E., Hutchings J., Jackson J., Kelley D., Kwok R., Layton C. (2015). Towards quantifying the increasing role of oceanic heat flux in sea ice loss in the new Arctic. Bull. Am. Meteorol. Soc..

[6] Polyakov I.V., Alkire M.B., Bluhm B.A., Brown K.A., Carmack E.C., Chierici M., Danielson S.L., Ellingsen I., Ershova E.A., Gårdfeldt K. (2020). Borealization of the Arctic Ocean in response to anomalous advection from sub-arctic seas. Front. Mar. Sci..

[7] Polyakov I.V., Pnyushkov A., Alkire M., Ashik I.M., Baumann T.M., Carmack E.C., Goszczko I., Guthrie J.D., Ivanov V.V., Kanzow T. (2017). Greater role for Atlantic inflows on sea-ice loss in the Eurasian Basin of the Arctic Ocean. Science.

[8] Oziel L., Massicotte P., Babin M., Devred E. (2022). Decadal changes in Arctic Ocean Chlorophyll *a*: Bridging ocean color observations from the 1980s to present time. Remote Sens. Environ..

[9] Ardyna M., Babin M., Gosselin M., Devred E., Rainville L., Tremblay J.-É. (2014). Recent Arctic Ocean sea ice loss triggers novel fall phytoplankton blooms. Geophys. Res. Lett..

[10] Renaut S., Devred E., Babin M. (2018). Northward expansion and intensification of phytoplankton growth during the early ice-free season in Arctic. Geophys. Res. Lett..

[11] Jakobsen T., Ozhigin V.K. (2011). The Barents Sea: Ecosystem, Resources, Management: Half a Century of Russian-Norwegian Cooperation.

[12] Matishov G.G., Dzhenyuk S.L., Denisov V.V., Zhichkin A.P., Moiseev D.V. (2012). Climate and oceanographic processes in the Barents Sea. Ber. Polarforsch..

[13] Loeng H., Drinkwater K. (2007). An overview of the ecosystems of the Barents Sea and Norwegian Seas and their response to climate variability. Deep-Sea Res. II.

[14] Sakshaug E., Johnsen G., Kovacs K. (2009). Ecosystem Barents Sea.

[15] ICES (2021). Working Group on the Integrated Assessments of the Barents Sea (WGIBAR). ICES Sci. Rep..

[16] ICES (2022). Working Group on the Integrated Assessments of the Barents Sea (WGIBAR). ICES Sci. Rep..

[17] Vinje T. (2001). Anomalies and trends of sea ice extent and atmospheric circulation in the Nordic Seas during the period 1864–1998. J. Climatol..

[18] Loeng H. (1991). Features of the physical oceanographic conditions in the central parts of the Barents Sea. Polar Res..

[19] Wassmann P., Reigstad M., Haug T., Rudels B., Carroll M.L., Hop H., Gabrielsen G.W., Falk-Petersen S., Denisenko S.G., Arashkevich E. (2006). Food webs and carbon flux in the Barents Sea. Progr. Oceanogr..

[20] Lind S., Ingvaldsen R.B., Furevik T. (2018). Arctic warming hotspot in the northern Barents Sea linked to declining sea-ice import. Nat. Clim. Change.

[21] Hunt G.L., Blanchard A.L., Boveng P., Dalpadado P., Drinkwater K.F., Eisner L., Hopcroft R.R., Kovacs K.M., Norcross B.L., Renaud P. (2013). The Barents and Chukchi Seas: Comparison of two Arctic shelf ecosystems. J. Mar. Syst..

[22] Kahru M., Brotas V., Manzano-Sarabia M., Mitchell B.G. (2011). Are phytoplankton blooms occurring earlier in the Arctic?. Glob. Chang. Biol..

[23] Arrigo K.R., van Dijken G.L. (2015). Continued increases in Arctic Ocean primary production. Prog. Oceanogr..

[24] Stige L.C., Dalpadado P., Orlova E., Boulay A.C., Durant J.M., Ottersen G., Stenseth N.C. (2006). Spatiotemporal statistical analyses reveal predator–driven zooplankton fluctuations in the Barents Sea. Progr. Oceanogr..

[25] Dalpadado P., Arrigo K.R., Hjøllo S.S., Rey F., Ingvaldsen R.B., Sperfeld E., van Dijken G.L., Stige L.C., Olsen A., Ottersen G. (2014). Productivity in the Barents Sea-Response to Recent Climate Variability. PLoS ONE.

[26] Dalpadado P., Ingvaldsen R., Hassel A. (2003). Zooplankton biomass variation in relation to climatic conditions in the Barents Sea. Polar Biol..

[27] Dvoretsky V.G., Dvoretsky A.G. (2013). Epiplankton in the Barents Sea: Summer variations of mesozooplankton biomass, community structure and diversity. Cont. Shelf Res..

[28] Dvoretsky V.G., Dvoretsky A.G. (2022). Coastal mesozooplankton assemblages during spring bloom in the eastern Barents Sea. Biology.

[29] Aarflot J.M., Skjoldal H.R., Dalpadado P., Skern-Mauritzen M. (2018). Contribution of *Calanus* species to the mesozooplankton biomass in the Barents Sea. ICES J. Mar. Sci..

[30] Eriksen E., Skjoldal H.R., Gjøsæter H., Primicerio R. (2017). Spatial and temporal changes in the Barents Sea pelagic compartment during the recent warming. Prog. Oceanogr..

[31] Poloczanska E.S., Burrows M.T., Brown C.J., Garcia Molinos J., Halpern B.S., Hoegh-Guldberg O., Kappel C.V., Moore P.J., Richardson A.J., Schoeman D.S. (2016). Responses of marine organisms to climate change across oceans. Front. Mar. Sci..

[32] McClain C.R. (2009). A decade of satellite ocean color observations. Annu. Rev. Mar. Sci..

[33] Gregg W.W., Conkright M.E. (2002). Decadal changes in global ocean chlorophyll. Geophys. Res. Lett..

[34] Gregg W.W., Rousseaux C.S. (2014). Decadal trends in global pelagic ocean chlorophyll: A new assessment integrating multiple satellites, *in situ* data, and models. J. Geophys. Res. Ocean..

[35] Lee Y.J., Matrai P.A., Friedrichs M.A.M., Saba V.S., Antoine D., Ardyna M., Asanuma I., Babin M., Belanger S., Benoît-Gagne M. (2015). An assessment of phytoplankton primary productivity in the Arctic Ocean from satellite ocean color/*in situ* chlorophyll-a based models. J. Geophys. Res. Ocean..

[36] Kalinka O.P., Vodopianova V.V. (2022). Aspects of satellite and subsatellite studies of chlorophyll-a in Arctic waters. IOP Conf. Ser. Earth Environ. Sci..

[37] Lewis K.M., Arrigo K.R. (2020). Ocean color algorithms for estimating chlorophyll a, CDOM absorption, and particle backscattering in the Arctic Ocean. J. Geophys. Res. Ocean..

[38] Matsuoka A., Hill V., Huot Y., Babin M., Bricaud A. (2011). Seasonal variability in the light absorption properties of western Arctic waters: Parameterization of the individual components of absorption for ocean color applications. J. Geophys. Res..

[39] Dalpadado P., Arrigo K.R., van Dijken G.L., Skjoldal H.R., Bagøien E., Dolgov A.V., Prokopchuk I.P., Sperfeld E. (2020). Climate effects on temporal and spatial dynamics of phytoplankton and zooplankton in the Barents Sea. Progr. Oceanogr..

[40] Makarevich P., Druzhkova E., Larionov V., Mahamane A. (2012). Primary producers of the Barents Sea. Diversity of Ecosystems.

[41] Makarevich P.R., Vodopianova V.V., Bulavina A.S. (2022). Dynamics of the spatial chlorophyll-a distribution at the Polar Front in the marginal ice zone of the Barents Sea during spring. Water.

[42] Makarevich P.R., Vodopianova V.V., Bulavina A.S., Vashchenko P.S., Ishkulova T.G. (2021). Features of the distribution of chlorophyll-a concentration along the western coast of the Novaya Zemlya archipelago in spring. Water.

[43] Strickland J.D.H., Parsons T.R. (1972). A Practical Handbook of Seawater Analysis.

[44] Aminot A., Rey F. (2000). Standard Procedure for the Determination of Chlorophyll a by Spectroscopic Methods.

[45] Parsons T.R., Maita Y., Lalli C.M. (1992). A Manual of Chemical and Biological Methods for Sea Water Analysis.

[46] Hurrell J.W., Kushnir Y., Ottersen G., Visbeck M. (2003). The North Atlantic Oscillation: Climate Significance and Environmental Impact.

[47] Hurrell J.W., Deser C. (2009). North Atlantic climate variability: The role of the North Atlantic oscillation. J. Mar. Syst..

[48] Jones P.D., Jónsson T., Wheeler D. (1997). Extension to the North Atlantic Oscillation using early instrumental pressure observations from Gibraltar and South-West Iceland. Int. J. Climatol..

[49] Matishov G., Zuyev A., Golubev V., Adrov N., Timofeev S., Karamusko O., Pavlova L., Fadyakin O., Buzan A., Braunstein A. (2004). Climatic Atlas of the Arctic Seas 2004: Part I. Database of the Barents, Kara, Laptev, and White Seas—Oceanography and Marine Biology.

[50] Ozhigin V., Ivshin V., Trofimov A., Karsakov A.L., Antsiferov M. (2016). The Barents Sea Water: Structure, Circulation, Variability.

[51] Norwegian Polar Institute (2022). Sea Ice Extent in the Barents Sea in September. Environmental Monitoring of Svalbard and Jan Mayen (MOSJ). http://www.mosj.no/en/climate/ocean/sea-ice-extent-barents-sea-fram-strait.html.

[52] Hastie T., Tibshirani R. (1987). Generalized additive models: Some applications. J. Am. Stat. Assoc..

[53] National Center for Atmospheric Research (2022) Climate Data Guide. https://climatedataguide.ucar.edu/climate-data.

[54] Stroeve J.C., Serreze M.C., Holland M.M., Kay J.E., Malanik J., Barrett A.P. (2012). The Arctic’s rapidly shrinking sea ice cover: A research synthesis. Clim. Change.

[55] Polyakov I.V., Bhatt U.S., Walsh J.E., Abrahamsen E.P., Pnyushkov A.V., Wassmann P.F. (2013). Recent oceanic changes in the Arctic in the context of long-term observations. Ecol. Appl..

[56] Alekseev G.V., Glok N.I., Vyazilova A.E., Kharlanenkova N.E., Kulakov M.Y. (2021). Influence of SST in Low Latitudes on the Arctic Warming and Sea Ice. J. Mar. Sci. Eng..

[57] Yashayaev I., Seidov D. (2015). The role of the Atlantic Water in multidecadal ocean variability in the Nordic and Barents Seas. Progr. Oceanogr..

[58] Stroeve J., Notz D. (2018). Changing state of Arctic sea ice across all seasons. Environ. Res. Lett..

[59] Engelsen O., Hegseth E., Hop H., Hansen E., Falk-Petersen S. (2002). Spatial variability of chlorophyll-*a* in the marginal ice zone of the Barents Sea, with relations to sea ice and oceanographic conditions. J. Mar. Syst..

[60] Wassmann P., Ratkova T., Andreassen I., Vernet M., Pedersen G., Rey F. (1999). Spring bloom development in the Marginal Ice Zone and the Central Barents Sea. Mar. Ecol..

[61] Degerlund M., Eilertsen H.C. (2010). Main Species Characteristics of Phytoplankton Spring Blooms in NE Atlantic and Arctic Waters (68–80 N). Estuar. Coast..

[62] Raymont J.E.G. (1980). Plankton and Productivity in the Oceans.

[63] Lewis K.M., Van Dijken G.L., Arrigo K.R. (2020). Changes in phytoplankton concentration now drive increased Arctic Ocean primary production. Science.

[64] Skagseth Ø., Furevik T., Ingvaldsen R., Loeng H., Mork K., Orvik K., Ozhigin V., Dickson R., Meincke J., Rhines P. (2008). Volume and heat transports to the Arctic Ocean via the Norwegian and Barents Seas. Arctic-Subarctic Ocean Fluxes.

[65] Ingvaldsen R.B. (2005). Width of the North Cape Current and location of the Polar Front in the western Barents Sea. Geophys. Res. Lett..

[66] Visbeck M., Chassignet E.P., Curry R., Delworth T., Dickson B., Krahman G., Hurrell J.W., Kushnir Y., Ottersen G., Visbeck M. (2003). The Ocean’s Response to North Atlantic Oscillation Variability. The North Atlantic Oscillation: Climate Significance and Environmental Impact.

[67] McGinty N., Guðmundsson K., Ágústsdóttir K., Marteinsdóttir G. (2016). Environmental and climactic effects of chlorophyll-a variability around Iceland using reconstructed satellite data fields. J. Mar. Syst..

[68] Arrigo K.R. (2014). Sea ice ecosystems. Ann. Rev. Mar. Sci..

[69] Ardyna M., Arrigo K. (2020). Phytoplankton dynamics in a changing Arctic Ocean. Nat. Clim. Change.

[70] Falk-Petersen S., Pavlov V., Berge J., Cottier F., Kovacs K.M., Lydersen C. (2015). At the rainbow’s end: High productivity fueled by winter upwelling along an Arctic shelf. Polar Biol..

[71] Wassmann P., Reigstad M. (2011). Future Arctic Ocean seasonal ice zones and implications for pelagic–benthic coupling. Oceanography.

[72] Oziel L., Neukermans G., Ardyna M., Lancelot C., Tison J.-L., Wassmann P., Sirven J., Ruiz-Pino D., Gascard J.-C. (2017). Role for Atlantic inflows and sea ice loss on shifting phytoplankton blooms in the Barents Sea. J. Geophys. Res. Oceans.

[73] Qu B., Gabric A.J. (2022). The multi-year comparisons of chlorophyll and sea ice in Greenland Sea and Barents Sea and their relationships with the North Atlantic Oscillation. J. Mar. Syst..

[74] Dong K., Kvile K.Ø., Stenseth N.C., Stige L.C. (2020). Associations among temperature, sea ice and phytoplankton bloom dynamics in the Barents Sea. Mar. Ecol. Prog. Ser..

[75] Sorteberg A., Kvingedal B. (2006). Atmospheric forcing on the Barents sea winter ice extent. J. Clim..

[76] Ding Q., Schweiger A., L’Heureux M., Battisti D., Po-Chedley S., Johnson N., Blanchard-Wrigglesworth E., Harnos K., Zhang Q., Eastman R. (2017). Influence of high-latitude atmospheric circulation changes on summertime Arctic sea ice. Nat. Clim. Chang..

[77] Bélanger S., Babin M., Tremblay J.É. (2013). Increasing cloudiness in Arctic damps the increase in phytoplankton primary production due to sea ice receding. Biogeosciences.

[78] Dvoretsky V.G., Dvoretsky A.G. (2022). Summer-fall macrozooplankton assemblages in a large Arctic estuarine zone (south-eastern Barents Sea): Environmental drivers of spatial distribution. Mar. Environ. Res..

[79] Dvoretsky V.G., Dvoretsky A.G. (2020). Arctic marine mesozooplankton at the beginning of the polar night: A case study for southern and south-western Svalbard waters. Polar Biol..

[80] Dvoretsky V.G., Dvoretsky A.G. (2018). Mesozooplankton in the Kola Transect (Barents Sea): Autumn and winter structure. J. Sea Res..

[81] Dvoretsky V.G., Dvoretsky A.G. (2021). Winter zooplankton in a small Arctic lake: Abundance and vertical distribution. Water.

[82] Dvoretsky V.G., Dvoretsky A.G. (2015). Early winter mesozooplankton of the coastal south-eastern Barents Sea. Estuar. Coast. Shelf Sci..

[83] Dvoretsky V.G., Dvoretsky A.G. (2023). Copepod assemblages in a large Arctic coastal area: A baseline summer study. Diversity.

[84] Dvoretsky V.G., Dvoretsky A.G. (2019). Summer macrozooplankton assemblages of Arctic shelf: A latitudinal study. Cont. Shelf Res..

