# Supplementary material for: Effects of Climate Change on Chlorophyll a in the Barents Sea: A Long-Term Assessment
Source: Biology (Basel). 2023 Jan 11;12(1):119. doi: 10.3390/biology12010119 (PMC9856002; doi:10.3390/biology12010119)
Supplement: Supplementary file 1 [file biology-12-00119-s001.zip › biology-2138150-supplementary.pdf]

Supplementary material for the paper
"Effects of Climate Change on Chlorophyll *a* in the Barents Sea: A Long-Term Assessment"
by
Vladimir G. Dvoretsky, Veronika V. Vodopianova and Aleksandra S. Bulavina

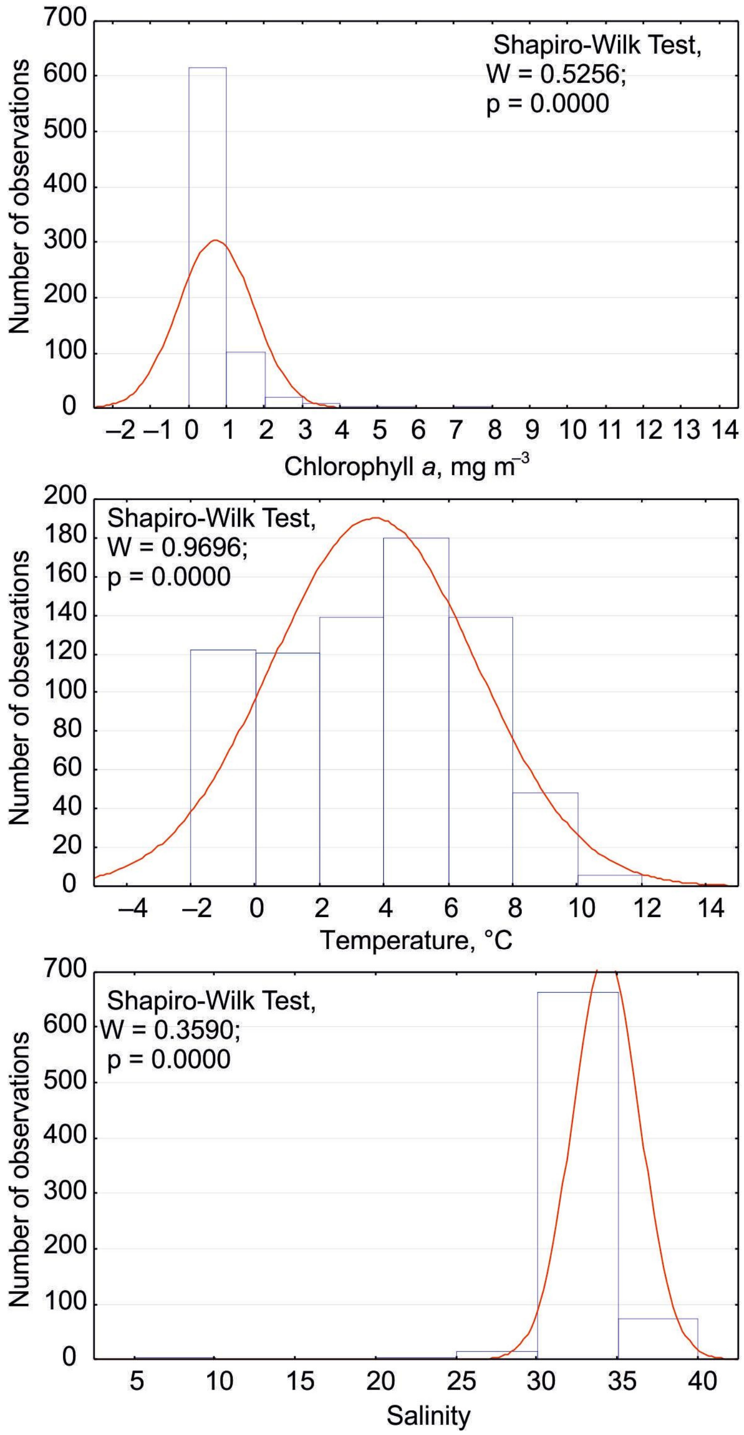

Figure S1. Histograms of data distributions (surface chlorophyll *a*, water temperature and salinity) in the Barents Sea in 1983–2021. Normality was tested with the Shapiro-Wilk test at α = 0.05.
